# Early clinical development of artemether-lumefantrine dispersible tablet: palatability of three flavours and bioavailability in healthy subjects

**DOI:** 10.1186/1475-2875-9-253

**Published:** 2010-09-03

**Authors:** Salim Abdulla, Baraka Amuri, Abdunoor M Kabanywanyi, David Ubben, Christine Reynolds, Steve Pascoe, Serge Fitoussi, Ching-Ming Yeh, Marja Nuortti, Romain Séchaud, Günther Kaiser, Gilbert Lefèvre

**Affiliations:** 1 Ifakara Health Institute, Ifakara and Dar es Salaam, Tanzania; 2 Medicines for Malaria Venture, Geneva, Switzerland; 3 Novartis Pharma Corporation, East Hanover, USA; 4 Novartis Pharma AG, Basel, Switzerland; 5 Mediscis, Lagord, France

## Abstract

**Background:**

Efforts to ease administration and enhance acceptability of the oral anti-malarial artemether-lumefantrine (A-L) crushed tablet to infants and children triggered the development of a novel dispersible tablet of A-L. During early development of this new formulation, two studies were performed in healthy subjects, one to evaluate the palatability of three flavours of A-L, and a second one to compare the bioavailability of active principles between the dispersible tablet and the tablet (administered crushed and intact).

**Methods:**

Study 1 was performed in 48 healthy schoolchildren in Tanzania. Within 1 day, all subjects tasted a strawberry-, orange- and cherry-flavoured oral A-L suspension for 10 seconds (without swallowing) in a randomized, single-blind, crossover fashion. The palatability of each formulation was rated using a visual analogue scale (VAS). Study 2 was an open, randomized crossover trial in 48 healthy adults given single doses of A-L (80 mg artemether + 480 mg lumefantrine) with food. The objectives were to compare the bioavailability of artemether, dihydroartemisinin (DHA) and lumefantrine between the dispersible tablet and the tablet administered crushed (primary objective) and intact (secondary objective).

**Results:**

Study 1 showed no statistically significant difference in VAS scores between the three flavours but cherry had the highest score in several ratings (particularly for overall liking). Study 2 demonstrated that the dispersible and crushed tablets delivered bioequivalent artemether, DHA and lumefantrine systemic exposure (area under the curve [AUC]); mean ± SD AUC_0-tlast _were 208 ± 113 vs 195 ± 93 h.ng/ml for artemether, 206 ± 81 vs 199 ± 84 h.ng/ml for DHA and 262 ± 107 vs 291 ± 106 h.μg/ml for lumefantrine. Bioequivalence was also shown for peak plasma concentrations (C_max_) of DHA and lumefantrine. Compared with the intact tablet, the dispersible tablet resulted in bioequivalent lumefantrine exposure, but AUC and C_max _values of artemether and DHA were 20-35% lower.

**Conclusions:**

Considering that cherry was the preferred flavour, and that the novel A-L dispersible tablet demonstrated similar pharmacokinetic performances to the tablet administered crushed, a cherry-flavoured A-L dispersible tablet formulation was selected for further development and testing in a large efficacy and safety study in African children with malaria.

## Background

In the treatment of uncomplicated malaria, there is an urgent need for alternative formulations that offer increased ease of administration, accuracy of dosing and compliance for infants and children [1], the main target population of artemisinin-based combination therapy (ACT) in Africa. In fact, for children almost all forms of ACT are available as crushed tablet only. The latter contrasts with the paediatric nature of the disease and the bitterness of ingredients. This was the impetus behind the development of a novel dispersible tablet formulation of artemether-lumefantrine (A-L), the most widely used ACT in Africa [2,3], ahead of the World Health Organization's call to make medicines child-friendly [4] and the recent recommendation to deploy ACT formulations appropriate for children [5].

In 2004, the A-L tablet (Coartem^® ^) became the first fixed-dose ACT to be prequalified by the World Health Organization, and received approval from the Food and Drug Administration in the US in April 2009 [6]. This tablet formulation, containing 20 mg of artemether and 120 mg of lumefantrine, has been proven to be efficacious and safe in the treatment of uncomplicated *Plasmodium falciparum *malaria when administered in a six-dose regimen for 3 days [7,8]. In terms of drug disposition, artemether and lumefantrine exhibit complementary pharmacokinetic characteristics. After oral administration, artemether is absorbed quickly, achieving maximum plasma concentrations (C_max_) after approximately 2 hours. It is rapidly and extensively demethylated to the pharmacologically active metabolite dihydroartemisinin (DHA). Both artemether and DHA exhibit short elimination half-lives of < 3 hours [9,10]. Lumefantrine is slowly absorbed reaching C_max _after approximately 6-8 hours, and is more slowly cleared with an elimination half-life of 3-6 days [10-12], thereby preventing recrudescence by destroying any residual parasites that remain after artemether and DHA have been cleared from the body [13]. Lumefantrine has never been used as a monotherapy and has therefore been protected from the development of parasite resistance unlike partner drugs used in other forms of ACT. Food intake significantly enhances the bioavailability of both artemether and lumefantrine, an effect which is far more pronounced for the lipophilic lumefantrine [13-15]. There is good evidence that a standard African diet is adequate to ensure optimal efficacy for A-L [16]. The pharmacokinetic features of artemether and lumefantrine are similar in children, when dosed according to their body weight, compared with adults [13].

When treating paediatric malaria patients, A-L tablets usually have to be crushed prior to administration to infants and young children. The crushed tablets, similarly to other anti-malarials, have a bitter taste that may cause children to spit out or vomit the drug, potentially resulting in underdosing [3] or in overdosing if re-dosed. A dispersible tablet containing the same amounts of artemether and lumefantrine like the standard tablet would allow a more accurate dosing of A-L and a simpler administration to sick children and infants compared with the tablet administered crushed [3]. As palatability is a contributing factor to compliance, particularly in children's medicine [17], the success of dispersible A-L in terms of acceptability to children would also be dependent on a flavour that masks the bitter taste of the drug [3].

Against this background, two studies were performed during early clinical development of A-L dispersible tablet. The first study evaluated the palatability of three flavours of A-L in healthy African schoolchildren (Study 1), followed by a second study which assessed the relative bioavailability of artemether, DHA and lumefantrine from A-L dispersible tablet compared with the tablet administered crushed (primary objective) and intact (secondary objective) in healthy European adults (Study 2). Study 1 was performed in schoolchildren rather than infants because palatability assessments in infants were considered not reliable. Furthermore, to standardize study conditions as much as possible, palatability was assessed at one centre in one country where malaria is endemic. For study 2, healthy adults were selected to avoid cumbersome blood collection in infants or children. The latter appeared justified given the similarity of artemether and lumefantrine pharmacokinetics between adults and children [13].

For convenience of the reader, methods and results are consecutively presented for study 1, followed by the respective sections for study 2.

## Methods

### Study 1: Palatability study

#### Study design

The palatability study was conducted at a primary school in Ifakara town, Tanzania, East Africa. Forty-eight healthy male and female children, aged 7-10 years, were enrolled, stratified by gender (24 girls and 24 boys). In the absence of previous tasting data on A-L, the sample size was based on practical considerations. The study protocol was reviewed and approved by the Independent Ethics Committee of the Ifakara Health Institute, Dar es Salaam, Tanzania, and written informed consent was obtained from the parents or legal guardians of the children before enrolment. For inclusion, the subjects had to be able to hold 2 ml of apple juice in their mouth for 10 seconds without swallowing and to complete a questionnaire. Exclusion criteria included any condition or dietary habit known to interfere with the sense of smell and taste, ingestion of any medication (except paracetamol) or significant illness within the previous two weeks, history of autonomic dysfunction, bronchospastic disease or atopic allergy, known hypersensitivity to any drug or artificial sweetener, and participation in any clinical investigation within the previous four weeks.

Three flavours of A-L were considered best in masking the bitter taste. The study was conducted similar to those previously reported [18,19]. In brief, the subjects received 2 ml of an oral suspension containing 20 mg of artemether and 120 mg of lumefantrine (strawberry-, orange- or cherry-flavoured) in a randomized, single-blind, crossover fashion. Study drug was administered into the mouth cavity using a 10 ml volume plastic syringe. The amount given represented half a treatment dose for children of this age. Following administration, the subject moved the study drug within the mouth cavity and then held it in the mouth for approximately 10 seconds. The study drug was not swallowed. All formulations had a yellow appearance to prevent any subject bias. The trial medication was provided as powder-in-bottle formulations; the oral suspension was prepared immediately prior to tasting. No food or beverage was allowed for two hours before the study commenced. The three administrations were performed within one day, separated by 45-minute intervals.

#### Determination of palatability

Immediately after each test dose, the child was asked to separately rate the flavour, smell, sweetness and overall liking of the medicine using a modified 100 mm visual analogue scale (VAS) that incorporated a facial hedonic scale [18,19]. The rating for overall liking was repeated at 2-5 minutes after the study drug had been spat out. In addition, 15-20 minutes after the last administration had been rated the children were asked which of the three administrations they thought tasted best (a ranking from 1 to 3 was performed). Any adverse events occurring during the study were recorded, with a final assessment at one hour following last drug administration. The VAS scores were analysed to determine whether a significant difference exists between flavours, using a SAS PROC MIXED procedure (e.g. using linear mixed effects modelling). The ranked data were analysed by Friedman's non-parametric procedure. All statistical tests employed a level of significance of 0.05.

## Results

### Study 1: Palatability study

All 48 randomized children completed the study. The mean age ± standard deviation (SD) was 8.6 ± 0.7 years. All study participants were of black ethnicity.

**Figure 1:**
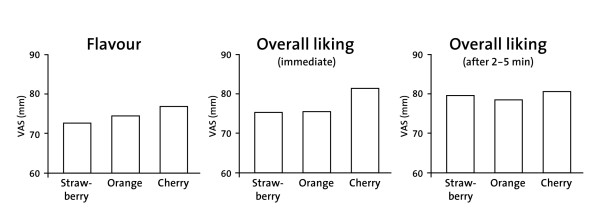
**Mean VAS palatability scores (24 girls, 24 boys)**. VAS = visual analogue scale. I did not like it = VAS score of 0 mm; I liked it very much = VAS score of 100 mm. As no significant gender difference was observed, data from girls and boys were pooled. There was no significant difference in pooled VAS scores between the three flavours for any rating (data not shown for smell and sweetness).

All flavours were highly rated with mean VAS scores ranging between 70 and 87 mm. As no significant gender difference was observed, data from girls and boys were pooled. There was no significant difference in pooled VAS scores between the three flavours for any rating. Numerically, cherry had the highest score in overall liking (immediately after administration and 2-5 minutes thereafter) and in the rating for flavour (Figure 1). The analysis of ranked data on the overall preference of a flavour also indicated the absence of a significant difference (*P *= 0.146).

Two mild adverse events (fever and clinical malaria) unrelated to study medication were reported which resolved upon treatment.

## Methods

### Study 2: Relative bioavailability study

#### Study design

This was an open-label, randomized, 3-period, 2-sequence, crossover study conducted at Mediscis, Lagord, France. A total of 48 healthy male and female adults between 18 and 50 years of age were recruited. The study protocol was approved by an Independent Ethics Committee (The Consultative Committee for the Protection of Persons in Biomedical Research, Poitiers Cedex, France). Subjects gave written informed consent to participate after being informed about the study. The three study treatments consisted of a single oral dose of A-L (4 dispersible tablets or 4 tablets of Coartem^®^, both corresponding in total to 80 mg of artemether and 480 mg of lumefantrine, a standard dose in patients), immediately (within 5 minutes) after consumption of a meal (FDA breakfast [20]). A-L was administered as: (A) 4 dispersible tablets following dispersion in water; (B) 4 tablets crushed and dispersed in water; or (C) 4 intact (uncrushed) tablets swallowed with water. Each subject received all 3 treatments. Treatments A and B were administered in study periods 1 and 2 in a randomized crossover fashion; Treatment C was always administered in study period 3. The treatments were separated by a 4-week washout.

#### Subjects and assessments

Subjects were judged healthy at screening (i.e. within three weeks before first dosing) on the basis of medical history, physical examination, vital signs measurements, electrocardiogram, laboratory assessments, negative urine drug and cotinine screens, and negative hepatitis and human immunodeficiency virus serologies. For inclusion, female subjects of childbearing age were required to use reliable methods of contraception during the study. Subjects were excluded if they had a history of autonomic dysfunction, bronchospastic disease, atopic allergy, or hypersensitivity to the study drug or related compounds. With the exception of contraceptives for females and drugs needed for adverse event treatment, no medication other than the study drug was allowed during the study.

Subjects were confined to the study center from 12-14 hours before until 48 hours after each drug administration. The study medication was prepared by a site pharmacist and administered immediately after a standard FDA breakfast by the study personnel as follows: Treatment A, fully dispersed in non-carbonated water; Treatment B, swallowed with non-carbonated water after crushing in a mortar to coarse particles; Treatment C, swallowed with non-carbonated water without previous crushing. For Treatments A and B, study drug and 40 ml of water were combined in a beaker followed by gentle shaking. After ingestion, another 40 ml of water was added to the beaker for rinsing; this additional volume was also consumed by the subject. In total, 240 ml of non-carbonated water were ingested for each treatment.

Safety assessments included physical examinations, electrocardiograms, vital signs measurements (blood pressure, pulse), and laboratory evaluations (biochemistry, hematology, urinalysis) prior to the study, at multiple times during the trial, and at study completion (last day of the last treatment period). The assessments during the study consisted of laboratory parameters (biochemistry, haematology) 24, 48, 72, 96 and 120 hours post-dose; vital signs at pre-dose as well as 24 and 48 hours thereafter, and an electrocardiogram pre-dose and 2, 8, 10, 12, 24, 36 and 48 hours post-dose. In addition, adverse event monitoring took place.

Blood samples for artemether and DHA analysis (3.5 ml) were collected before drug intake and at 0.5, 0.75, 1, 1.5, 2, 3, 4, 6, 8, 10, 12, 16, 24, 36 and 48 hours after dosing. For lumefantrine measurements, blood sampling (2.5 ml) occurred at pre-dose and at 0.5, 1, 1.5, 2, 3, 4, 5, 6, 8, 10, 12, 16, 24, 36, 48, 72, 96, 120, 168, 216 and 264 hours post-dose. All blood samples were taken by venepuncture into heparin-coated tubes. After centrifugation, aliquots of plasma were taken and frozen at -70°C until analysis.

#### Bioanalytics

Artemether and DHA were measured in plasma using a reversed-phase high-performance liquid chromatography (HPLC) with atmospheric pressure chemical ionization (APCI) and mass spectrometry (MS) detection (with modifications from [21]); the limit of quantification (LOQ) was 5.0 ng/ml for both analytes. Lumefantrine was measured in plasma by HPLC with tandem mass spectrometry (MS/MS) detection; the LOQ was 50 ng/ml. The within-study assay validation showed an assay precision (percent coefficient of variation [%CV]) of 3.8 to 6.3%, with a deviation (bias) of -4.7 to 5.0% of nominal concentrations (0.1, 2.0 and 16.0 μg/ml).

#### Pharmacokinetic and statistical evaluation

Artemether, DHA and lumefantrine plasma concentration-time profiles were analysed by standard non-compartmental methods using WinNonlin Pro, Version 5.0.1, Pharsight Corporation, Mountain View, CA, USA. The following pharmacokinetic parameters were determined: C_max_, t_max _(time of C_max_), area under the curve calculated with the linear trapezoidal method from 0 h to the time of the last quantifiable plasma concentration (AUC_0-tlast_), AUC extrapolated to time infinity (AUC_0-inf_) and terminal elimination half-life (t1/2). The slope of the linear regression analysis from the last three (or more) log concentration-time points was used to determine the terminal elimination rate constant (λ_z_) and t1/2 (t1/2 = ln2/λ_z_).

Logarithmic-transformed C_max_, AUC_0-tlast _and AUC_0-inf _values were compared using linear mixed-effects models with sequence, period and treatment as fixed effects and subject nested in sequence as random effect. The treatment difference estimate on the log-scale and its 90% confidence interval (CI) were calculated; these estimates were back-transformed, and the resulting ratio and its 90% CIs were provided. Although it was not the intention of the study to prove bioequivalence between treatments, the 90% CIs were compared with the standard bioequivalence range of 0.80-1.25 [22]. The comparison of artemether, DHA and lumefantrine exposure after administration of the dispersible tablet (Treatment A) versus the tablet administered crushed (Treatment B) was the primary study objective, with lumefantrine AUC prospectively defined as the key study endpoint. Comparisons between the dispersible tablet and intact tablet (Treatment C) were secondary objectives.

Based on an intra-subject variability of 44% for lumefantrine AUC assessed in a previous study with Coartem^® ^(Novartis, data on file), a sample size of 48 subjects was calculated. This ensured at least 80% power to obtain a 90% CI for lumefantrine AUC within 0.77 and 1.30 [23].

## Results

### Study 2: Relative bioavailability study

All 48 randomized subjects (22 females, 26 males) completed the study. The mean age ± SD was 33.1 ± 7.8 years and mean weight was 68.3 ± 9.8 kg. Most study participants (98%) were Caucasian, one subject was of mixed ethnicity (Black-Caucasian).

**Figure 2:**
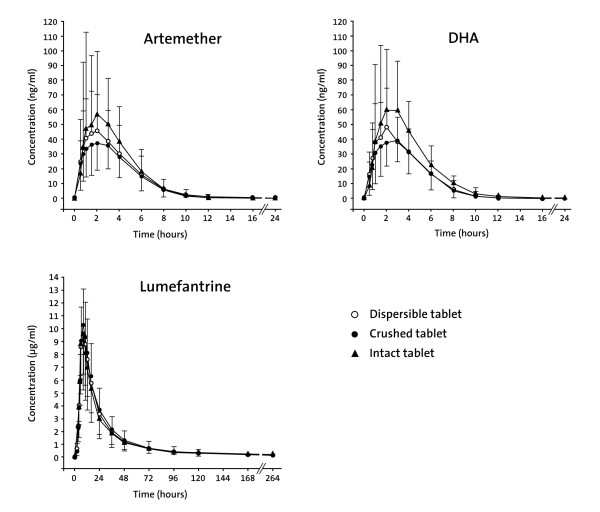
**Mean plasma concentration-time profiles (linear scale) of artemether, dihydroartemisinin and lumefantrine in healthy subjects (n = 48)**. DHA = dihydroartemisinin. A single oral dose of 80 mg of artemether and 480 mg of lumefantrine (4 tablets) was administered to healthy adults as dispersible tablet (Treatment A, fully dispersed in water), crushed tablet (Treatment B, swallowed with water after crushing to coarse particles) or intact tablet (Treatment C, swallowed with water without previous crushing). Bars represent one standard deviation.

Pharmacokinetic parameters of artemether, DHA and lumefantrine and results of statistical analysis are summarized in Tables 1 and 2, respectively. The corresponding mean plasma concentration-time profiles are shown in Figure 2.

**Table 1:** Relative bioavailability study - pharmacokinetic parameters of artemether, dihydroartemisinin and lumefantrine in healthy subjects (n = 48)

	Treatment A dispersible tablet	Treatment B crushed tablet	Treatment C intact tablet
**Artemether**			
t_max _(hours)	2.0 (0.5-6.0)	2.0 (0.5-6.0)	2.0 (0.8-6.0)
C_max _(ng/ml)	58.4 ± 32.2	48.0 ± 22.2	83.8 ± 59.7
AUC_0-tlast _(h.ng/ml)	208 ± 113	195 ± 93	259 ± 150
AUC_0-inf _(h.ng/ml)	281 ± 120 (n = 24)*	261 ± 116 (n = 20)*	330 ± 158 (n = 33)*
t_1/2 _(hours)	2.2 ± 1.5 (n = 29, 60%)*	2.7 ± 2.2 (n = 25, 52%)*	2.3 ± 1.9 (n = 36, 75%)*
**Dihydroartemisinin**			
t_max _(hours)	2.0 (0.8-6.0)	2.5 (1.0-8.0)	2.0 (0.8-6.0)
C_max _(ng/ml)	57.3 ± 24.9	50.0 ± 18.9	90.4 ± 48.9
AUC_0-tlast _(h.ng/ml)	206 ± 81	199 ± 84	285 ± 98
AUC_0-inf _(h.ng/ml)	266 ± 80 (n = 26)*	261 ± 84 (n = 25)*	326 ± 103 (n = 38)*
t_1/2 _(hours)	2.1 ± 0.9 (n = 28, 58%)*	2.2 ± 1.1 (n = 27, 56%)*	2.3 ± 1.5 (n = 39, 81%)*
**Lumefantrine**			
t_max _(hours)	8.0 (6.0-12.0)	8.0 (6.0-12.0)	8.0 (5.0-12.0)
C_max _(μg/ml)	9.9 ± 3.0	10.8 ± 2.8	9.8 ± 4.2
AUC_0-tlast _(h.μg/ml)	262 ± 107	291 ± 106	243 ± 117
AUC_0-inf _(h.μg/ml)	279 ± 106 (n = 46)*	316 ± 119 (n = 47)*	281 ± 133 (n = 40)*
t_1/2 _(hours)	118 ± 55 (n = 46, 96%)*	115 ± 32 (n = 47, 98%)*	119 ± 51 (n = 41, 85%)*

*Reduced sample size because log-linear regression analysis did not allow for a proper assessment of the terminal elimination rate constant (adjusted r^2 ^< 0.75) or extrapolated area contributed > 20% of the total AUC_0-inf_.

Data are median (range) for t_max _and mean ± SD for all other parameters. A single oral dose of 80 mg of artemether and 480 mg of lumefantrine was administered.

**Table 2:** Relative bioavailability study - statistical analysis

	Parameter	Ratio (90% CI)*
***Treatment A (dispersible tablet) vs B (crushed tablet) (primary analysis)***		
Artemether	AUC_0-inf _AUC_0-tlast _C_max_	0.94 (0.86-1.02) 1.03 (0.94-1.13) 1.17 (1.06-1.29)
Dihydroartemisinin	AUC_0-inf _AUC_0-tlast _C_max_	1.05 (0.99-1.11) 1.03 (0.93-1.15) 1.14 (1.04-1.24)
Lumefantrine	AUC_0-inf _AUC_0-tlast _C_max_	0.90 (0.85-0.95) 0.89 (0.84-0.94) 0.91 (0.86-0.96)
***Treatment A (dispersible tablet) vs C (intact tablet) (secondary analysis)***		
Artemether	AUC_0-inf _AUC_0-tlast _C_max_	0.80 (0.71-0.90) 0.80 (0.73-0.88) 0.73 (0.65-0.82)
Dihydroartemisinin	AUC_0-inf _AUC_0-tlast _C_max_	0.78 (0.72-0.84) 0.69 (0.62-0.77) 0.65 (0.58-0.73)
Lumefantrine	AUC_0-inf _AUC_0-tlast _C_max_	1.06 (0.96-1.16) 1.12 (1.02-1.23) 1.07 (0.97-1.18)

*Ratio (90% CI) = ratio of the geometric means and 90% confidence interval for the comparison of Treatments A vs B and Treatments A vs C, respectively.

A single oral dose of 80 mg of artemether and 480 mg of lumefantrine (4 tablets) was administered to healthy adults as follows: Treatment A, fully dispersed in water; Treatment B, swallowed with water after crushing to coarse particles; Treatment C, swallowed with water without previous crushing.

All artemether and DHA pre-dose concentrations were below the LOQ indicating the absence of any carry-over effect between study periods. In a number of subjects, log-linear regression analysis did not allow for a proper assessment of the terminal elimination rate constant (adjusted r^2 ^< 0.75). Hence, associated parameters (i.e. t1/2 and AUC_0-inf_) could not always be determined. For lumefantrine, 5 pre-dose concentrations were above the LOQ (range, 0.05 to 0.11 μg/ml) in study period 2. In period 3, 12 pre-dose concentrations were above the LOQ (range, 0.05 to 0.14 μg/ml). However, all of these pre-dose concentrations were ≤ 2.7% of C_max _in the respective plasma concentration-time profile, indicating the absence of a relevant carry-over effect [22].

### Dispersible tablet versus tablet administered crushed

Following administration of A-L as dispersible tablet (Treatment A) and crushed tablet (Treatment B), artemether, DHA and lumefantrine pharmacokinetics were similar. Standard bioequivalence criteria (90% CIs for the ratio of geometric means within 0.80 and 1.25) were met for AUC_0-tlast _and AUC_0-inf _of artemether, DHA and lumefantrine. Likewise, the 90% CIs for DHA and lumefantrine C_max _also met the standard bioequivalence criterion. Mean C_max _of artemether was 17% higher with the dispersible tablet compared to the tablet administered crushed, and the upper boundary of the equivalence range was marginally exceeded (Table 2). For all analytes, median t_max _was virtually identical between the dispersible tablet and the tablet administered crushed. No notable difference in t1/2 was observed.

### Dispersible tablet versus tablet administered intact

After administration of A-L as dispersible tablet (Treatment A) and intact (uncrushed) tablet (Treatment C), some differences in artemether and DHA pharmacokinetics were observed, while bioequivalence criteria were met for lumefantrine AUC_0-tlast_, AUC_0-inf _and C_max _(Table 2). Exposure values (AUC_0-tlast _and AUC_0-inf_) for artemether and DHA were 20% and 22-31%, respectively, lower with the dispersible tablet compared to the intact tablet, with 90% CIs not including unity and being outside the bioequivalence range (Table 2). Likewise, administration of A-L as dispersible tablet resulted in a 27% (artemether) and 35% (DHA) lower C_max _compared to treatment with intact tablet. For all analytes, median t_max _and mean t1/2 were comparable between the dispersible tablet and the tablet administered intact (i.e. uncrushed).

### Tablet administered crushed versus tablet administered intact

The comparison of the tablet administered crushed (Treatment B) and the intact tablet (Treatment C) was not prospectively defined in the study protocol, and no statistical test was performed. Nevertheless, upon inspection of arithmetic means (Table 1), it is apparent that there were notable differences in the exposure to artemether and DHA between the crushed and intact tablets, with about 20 to 45% lower AUC_0-tlast_, AUC_0-inf _and C_max _values for the tablet administered crushed. The pharmacokinetics of lumefantrine was similar for the two treatments. Overall, the descriptive differences in artemether and DHA bioavailability parameters observed between Treatments B and C were comparable to those seen between Treatments A and C.

### Clinical observations

Single doses of A-L given as dispersible tablets and tablets (crushed or intact) were overall well tolerated. Thirty-two subjects reported 75 adverse events during the study without any clinically relevant difference between treatments. Fewer subjects reported adverse events in Treatment group C (intact tablets), most likely due to the absence of a bitter taste caused by dispersed or crushed A-L tablets. The most commonly reported adverse event was mild-to-moderate headache for all three treatments. One subject in Treatment group B (crushed tablets) reported severe cystitis, which was considered unrelated to study medication; the event resolved upon antibiotic treatment. There were no clinically relevant changes in clinical laboratory parameters, electrocardiograms or vital signs over the study course.

## Discussion

During early clinical development of A-L dispersible tablet, one study investigated the palatability of three flavours of A-L in healthy African schoolchildren using an oral suspension formulation prepared immediately prior to tasting from powder-in-bottle suspended with water. Another study assessed the relative bioavailability of artemether, DHA and lumefantrine from the novel A-L dispersible tablet formulation compared with the tablet (crushed and intact) in healthy European adults. The principal findings of these two studies can be summarized as follows: (1) All three tested flavours (strawberry, orange and cherry) appeared appropriate to improve the taste of A-L but cherry was overall preferred by children; (2) exposure to artemether, DHA and lumefantrine was comparable for the dispersible and crushed tablet formulations in healthy adults; and (3) there were differences of the same magnitude in the exposure to artemether and DHA between the dispersible tablet and the intact tablet, and between the crushed and intact tablets in adult healthy subjects, while exposure to lumefantrine was similar in these comparisons.

The cherry flavour appeared appropriate to assure a high acceptability of A-L dispersible tablet in small infants and children. However, caveats to this conclusion are the small sample size and that the children investigated were healthy schoolchildren, which may limit the generalizability of results from this palatability study to other populations from other areas. Nevertheless, the cherry flavour was selected for further clinical development of the A-L dispersible tablet. Even though the cherry fruit is not native to Africa, it is often found in soft drinks and other medications such as antibiotic syrups [3]. As such, this flavour is familiar to African children.

Following the palatability study, the pharmacokinetic performance of the A-L dispersible tablet was investigated in a relative bioavailability study. So far, the crushed tablet was the usual way to administer A-L to infants and children, and dosing recommendations for the paediatric population were originally made accordingly [3]. Therefore, the primary objective of the relative bioavailability study was the comparison between the novel A-L dispersible tablet and the crushed tablet in healthy subjects before embarking on a large efficacy and safety study in children with malaria. Although it was not the intention of the study to formally prove bioequivalence between treatments, the results showed that the dispersible and crushed tablets delivered bioequivalent artemether, DHA and lumefantrine exposure (AUC); bioequivalence was also shown for DHA and lumefantrine C_max_. The slightly increased artemether C_max _(17%) following administration of A-L dispersible tablet is judged clinically irrelevant considering the background variability of this parameter. The usefulness of the dispersible tablet has been subsequently confirmed in a multicenter, investigator-blinded, randomized, non-inferiority study which compared the efficacy and safety of A-L dispersible tablet and the tablet administered crushed in 899 African infants and children with uncomplicated falciparum malaria. The dispersible formulation was as efficacious as the crushed tablet, and had a similar safety profile [24]. Within that study, the similarity of artemether, DHA and lumefantrine pharmacokinetics between the dispersible and the crushed tablet formulations has been confirmed in the target population [13,24]. Hence, the transition to the new dispersible tablet of A-L is not thought to present any significant challenges as the dosing pattern is the same as for the tablet administered crushed. In fact, the A-L dispersible tablet has been approved already by more than 20 countries in Africa, with more countries to follow in the near future [3].

The statistical comparison of the plasma exposure parameters between the A-L dispersible tablet and the intact tablet yielded significantly lower AUC and C_max _values for artemether (by 20% and 27%, respectively) and DHA (by 22-31% and 35%, respectively) following intake of the dispersible tablet. Similar descriptive differences were seen between the crushed and intact tablet. Exposure to lumefantrine, however, was similar for the three treatments. Hence, it appears that administering A-L as an oral suspension (prepared from either the dispersible tablet or the crushed tablet) had an impact on artemether and DHA bioavailability, but not on lumefantrine. The pharmacokinetic results obtained for the intact tablet were in accordance with a previous study in healthy subjects, which used an identical administration and blood sampling design [12,25]. *In vitro *dissolution profiles of the dispersible tablet and both the crushed and intact tablets (from the same batches as used in the present study) were similar for artemether and lumefantrine (Novartis, data on file). Loss of study drug during preparation or administration of study medication can be excluded given the highly standardized procedures applied, and also because no decrease in lumefantrine exposure was observed. There is no proven explanation for the observed differences in relative bioavailability of artemether and its metabolite DHA between the suspension (dispersible or crushed tablet) and the intact tablet. However, as artemether is known to be chemically unstable under acidic conditions [26], it can be hypothesized that the extent of degradation in the stomach may differ between a solid dosage form and a suspension. As acid-catalyzed degradation of artemether cannot occur until the drug enters solution and the dissolution process is expected to take longer for the intact tablet than for the suspension, a larger fraction of the suspension dose as compared to the intact tablet dose may be expected to decompose. This may finally result in a reduced absorption and lower bioavailability of artemether. The observation that C_max _and AUC of artemether and its metabolite DHA vary in the same proportions supports this hypothesis. The slightly lower exposure to artemether and DHA, however, had no impact on clinical outcomes as demonstrated in a large clinical trial [24]. The dispersible tablet and the tablet administered crushed were both highly effective and appeared well tolerated and safe, consistent with historical data gathered with the intact tablet [9,27-29].

## Conclusions

The data of these two studies suggested that cherry is a suitable flavour for the development of an A-L dispersible tablet, and demonstrated that the pharmacokinetic characteristics of artemether, DHA and lumefantrine are similar for the dispersible and crushed A-L tablet formulations in adult healthy volunteers. Thus, a cherry-flavoured A-L dispersible tablet was selected for further clinical development in patients.

## Competing interests

Christine Reynolds, Steve Pascoe, Ching-Ming Yeh, Marja Nuortti, Romain Séchaud, Günther Kaiser and Gilbert Lefèvre are employees of Novartis Ltd.

## Authors' contributions

SA, BA and AMK participated in the design of study 1 and supervised the enrolment of subjects. DU participated in planning and design of studies 1 and 2. CR and CMY participated in the design of study 1 and coordinated data entry, collection and analysis of its data. SP participated in data entry and collection of data related to study 2. SF participated in the design of study 2 and supervised the enrolment of healthy adult subjects. MN performed the statistical analysis of study 2. RS, GK and GL participated in the design of study 2 and did the pharmacokinetic analyses.

All authors assisted with data interpretation and participated in the preparation of the manuscript. All authors read and approved the final manuscript.
